# The Role of the Vaginal Microbiome in Gynecological Diseases: Mechanistic Insights and Emerging Interventions

**DOI:** 10.3390/biology15050432

**Published:** 2026-03-05

**Authors:** Yiming Zhang, Tiantian Wei, Changying Zhao, Lei Zhang

**Affiliations:** 1 Microbiome-X, School of Public Health, Cheeloo College of Medicine, Shandong University, Jinan 250012, China; 2 School of Pharmaceutical Engineering, Jining Medical University, Jining 272073, China

**Keywords:** vaginal microbiome, vaginal dysbiosis, *Lactobacillus*, gynecological diseases, sexually transmitted infections, probiotics

## Abstract

The vaginal microbiome (VM), a complex and dynamic microbial ecosystem, is now recognized as a central determinant of female reproductive and gynecologic health. Under homeostatic conditions, a *Lactobacillus*-dominant ecosystem maintains vaginal acidity, provides colonization resistance, and modulates mucosal immunity. Conversely, vaginal dysbiosis—characterized by *Lactobacillus* depletion and anaerobic or aerobic overgrowth—is associated with infectious vaginitis, increased susceptibility to sexually transmitted infections, and non-infectious conditions such as genitourinary syndrome of menopause. This review provides an integrated overview of the composition, functional characteristics, and host interactions of the VM across health and disease. We highlight major mechanisms by which microbial dysbiosis contributes to disease pathogenesis, including biofilm formation, altered microbial metabolism, and immune dysregulation. In addition, we discuss the translational potential of the VM as a source of diagnostic and prognostic biomarkers and as a target for emerging microbiome-dependent therapeutic strategies. Collectively, current evidence supports the view that vaginal dysbiosis is a heterogeneous and context-dependent state driven by distinct pathogen- and host-related mechanisms, underscoring the importance of prioritizing microbiome restoration rather than pathogen eradication alone.

## Simple Summary

Recent studies reveal that the vaginal microbiome plays a crucial role in maintaining reproductive health and preventing disease. In healthy women, the microbiome is predominantly composed of *Lactobacillus* species, which help prevent infections and support normal immune function. When this microbial balance is disrupted, a condition known as microbial dysbiosis, women may develop common vaginal disorders such as bacterial vaginosis and candidiasis, and may also become more vulnerable to sexually transmitted infections. This review summarizes the changes in the vaginal microbiome across different disease states, its impact on vaginal health, and the mechanisms through which microbial imbalances influence disease progression. We also discuss promising strategies, including the use of probiotics and microbiome-based therapies, that aim to restore a healthy vaginal environment. Understanding these processes may help guide the development of more personalized and effective strategies for improving women’s health.

## 1. Introduction

The human microbiome, comprising bacteria, fungi, viruses, and archaea, is integral to host physiology, metabolism, and immunity across various body sites [1,2]. Among these mucosal ecosystems, the vaginal microbiome (VM) occupies a unique and critical niche, playing a paramount role in safeguarding reproductive health and overall well-being [3]. Historically viewed through the lens of specific pathogens, our understanding has evolved to recognize vaginal health as a state of ecological balance, primarily maintained by beneficial *Lactobacillus* species which produce lactic acid, antimicrobial compounds, and immunoregulatory metabolites [4,5].

Advancements in high-throughput sequencing have revolutionized the characterization of the VM. It has revealed distinct community state types (CSTs), ranging from *Lactobacillus*-dominant CSTs (I, II, V), typically characterized by *Lactobacillus crispatus*, *L. gasseri*, or *L. jensenii*, to anaerobe-rich CST IV enriched in *Gardnerella* and other strict anaerobes associated with dysbiosis and disease [6]. This microbial equilibrium is regulated by host factors, including hormonal fluctuations, genetics, and behavior [7,8,9]. These community configurations are not merely taxonomic classifications; rather, they represent distinct functional states that govern disease susceptibility. Deviation from this balance—vaginal dysbiosis—is not a mere bystander but a key driver in the pathophysiology of highly prevalent conditions. Emerging evidence further suggests that patterns of dysbiosis and disease susceptibility vary across life stages, including prepubertal, reproductive, and postmenopausal periods, underscoring the importance of hormonal and developmental context in interpreting microbiome–disease associations. Vaginal dysbiosis underpins bacterial vaginosis (BV), alters susceptibility to and clearance of viral pathogens like human papillomavirus (HPV) and human immunodeficiency virus (HIV), and contributes to the inflammatory milieu of aerobic vaginitis (AV) and vulvovaginal candidiasis (VVC) [10,11,12]. Furthermore, the VM’s role extends to non-infectious contexts, such as the genitourinary syndrome of menopause (GSM) and polycystic ovarian syndrome (PCOS), where host physiological and hormonal alterations are closely intertwined with microbial instability [13,14].

The clinical implications of these insights are profound. The limitations of conventional antimicrobial therapies—namely high recurrence rates and collateral damage to beneficial flora—have necessitated the development of microbiome-centric strategies. [15,16]. This includes the development of probiotics containing protective *Lactobacillus* strains and the exploratory application of vaginal microbiota transplantation (VMT) for refractory cases as strategies to restore functional community stability [17,18,19]. In addition, multi-omics platforms are increasingly applied for biomarker discovery and personalized therapeutic guidance.

This review provides a comprehensive synthesis of the vaginal microbiome’s pivotal role at the intersection of microbial ecology, immunology, and clinical gynecology. We detail the composition and determinants of a healthy VM, systematically examine the consequences and mechanisms of its disruption across a range of infectious and non-infectious diseases, and evaluate the diagnostic and therapeutic innovations emerging from this knowledge. By framing vaginal health through an ecological lens, we underscore the transformative potential of moving from pathogen eradication to microbiome restoration, paving the way for more precise, effective, and sustainable interventions in women’s healthcare.

## 2. Vaginal Microbial Community

The VM represents a unique mucosal ecosystem that plays a central role in maintaining reproductive and gynecologic health [20]. In healthy reproductive-aged women, this ecosystem is typically dominated by *Lactobacillus* species, which maintain an acidic vaginal environment and contribute to pathogen exclusion, immune modulation, and mucosal barrier integrity through the production of lactic acid, antimicrobial compounds, and immunoregulatory metabolites, thereby shaping local innate and adaptive immune responses [3].

However, the VM is not static and undergoes substantial fluctuations across the menstrual cycle, pregnancy, and the lifespan, and is shaped by hormonal, behavioral, and host genetic factors. Increasing recognition of the VM’s functional importance has driven efforts to characterize its taxonomic composition, ecological structure, and determinants. In the following sections, we summarize current knowledge on the composition, community state types, and host factors that influence the VM, providing a foundation for understanding its role in vaginal health and disease.

### 2.1. Characteristics of the Vaginal Microbiome

The VM comprises a complex assemblage of bacteria, fungi, protozoa, and viruses [21], forming a dynamic mucosal ecosystem that is highly responsive to hormonal fluctuations across the female lifespan [10,22]. This ecosystem plays a central role in maintaining female reproductive health by preventing pathogenic colonization, regulating mucosal immune responses, and preserving epithelial barrier integrity [5,23,24,25].

In healthy reproductive-age women, this ecosystem is characterized by low microbial diversity and dominance of *Lactobacillus* species, most notably *L. crispatus*, *L. jensenii*, *L. gasseri*, and *L. iners* [10,26,27]. These *Lactobacillus* maintain an acidic vaginal environment (pH < 4.5) primarily through the production of D- and L-lactic acid, as well as hydrogen peroxide (H_2_O_2_) and bacteriocins, thereby suppressing pathogen overgrowth, shaping local immune responses, and supporting epithelial barrier integrity [28,29].

Among them, *L. crispatus* is widely regarded as the most protective, owing to its robust lactic acid production, stable colonization, and sustained output of antimicrobial metabolites [28,30]. In contrast, *L. iners*, although frequently detected in women with clinically normal vaginal microbiota, is commonly associated with transitional microbial states and increased susceptibility to dysbiosis. Unlike *L. crispatus*, *L. iners* primarily produces L-lactic acid, which is less effective in pathogen suppression than D-lactic acid [4,31]. Moreover, it encodes inerolysin, a pore-forming toxin that may compromise epithelial barrier integrity and facilitate pathogen colonization [32]. These functional differences help explain why *L. iners* dominated communities may be less stable and more susceptible to infection.

### 2.2. Community State Types of the Vaginal Microbiome

**Figure 1:**
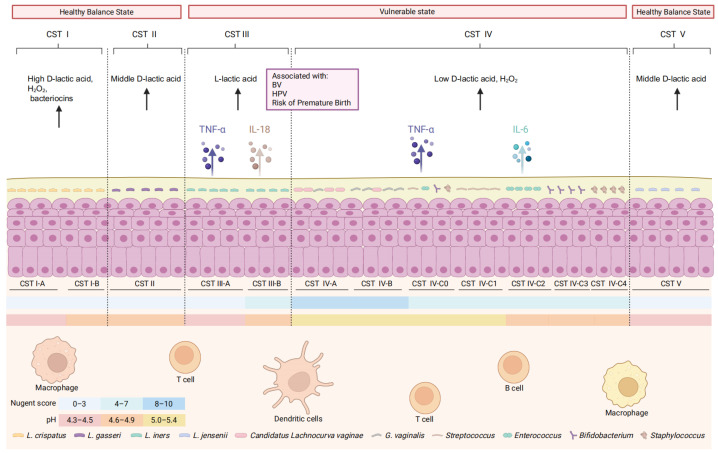
Vaginal microbiome CST and their associations with health and disease. *Lactobacillus*-dominated CSTs are generally associated with vaginal homeostasis, whereas CST IV, characterized by reduced *Lactobacillus* abundance and increased microbial diversity, is linked to elevated vaginal pH, enhanced inflammation, and an increased risk of gynecological disorders. The figure also depicts representative host immune cell populations in the vaginal mucosa. Inflammatory cytokines (TNF-α, IL-18, IL-6) represent key inflammatory factors associated with different CSTs. Black arrows indicate the secretion of metabolites by bacteria, while black dashed lines show the boundaries between different CSTs.

The VM exhibits substantial inter-individual variation. To systematically describe this ecological variation within the vaginal microbiota, researchers have developed the CST framework, which classifies vaginal microbial communities based on their dominant taxa and ecological features. The original CST model, derived from 16S rRNA gene sequencing of vaginal samples from North American women, identified five major CSTs: CST I (*L. crispatus* dominated), CST II (*L. gasseri* dominated), CST III (*L. iners* dominated), CST IV (diverse anaerobes dominate including *Gardnerella*, *Prevotella*, *Sneathia*, and *Atopobium*), and CST V (*L. jensenii* dominated) (Figure 1) [5].

CSTs I, II and V are generally associated with vaginal health and stability, reflecting *Lactobacillus*-dominated ecosystems with low microbial diversity and an acidic vaginal environment. In contrast, CST III and particularly CST IV are more frequently associated with vaginal dysbiosis. Longitudinal studies have demonstrated that the vaginal microbiome is highly dynamic, and women may transition between CSTs across the lifespan in response to hormonal fluctuations, sexual activity, antibiotic exposure, and other environmental or host-related factors. Concurrently, CST III is often observed as a transitional state preceding shifts toward CST IV, consistent with its intermediate ecological position and reduced metabolic and protective capacity compared with *L. crispatus*-dominated communities [33].

CST IV is characterized by higher microbial diversity, reduced *Lactobacillus* abundance, elevated vaginal pH (>5.0), and increased inflammatory signatures. This community state has been consistently linked to BV, increased susceptibility to STIs, and adverse pregnancy outcomes such as preterm birth. Importantly, potentially opportunistic microorganisms—including *G. vaginalis*, *Escherichia coli*, *Group B Streptococcus* (GBS), *Mycoplasma genitalium*, and *Candida albicans*—can be detected in asymptomatic women. Their pathogenic potential is strongly influenced by the surrounding microbial community structure and host immune status.

The levels of vaginal microbiota in different vaginal diseases are summarized in Table 1. Overall, several cross-disease patterns can be observed. Most infectious vaginal conditions are characterized by a reduction in *Lactobacillus* species—particularly *L. crispatus*—accompanied by the enrichment of specific anaerobic or facultative pathogens. BV and TV show prominent increases in anaerobic genera such as *Gardnerella*, *Prevotella*, and *Atopobium*, whereas AV is more frequently associated with aerobic bacteria including *Escherichia*, *Streptococcus*, and *Staphylococcus*. In contrast, VVC is distinguished by fungal overgrowth, while bacterial community changes are comparatively variable. STI-related conditions often occur in the context of reduced *Lactobacillus* dominance and increased microbial diversity, although pathogen-specific patterns are evident. These trends indicate that vaginal diseases are generally associated with shifts in overall community structure rather than uniform changes in individual taxa, underscoring the importance of ecological context in interpreting microbial abundance patterns.

Recent advances in metagenomic sequencing and unsupervised clustering have expanded this classification to as many as 13 CST subtypes, providing finer resolution of microbial structure and function [3,6]. However, despite its utility in microbial stratification, the CST framework may not fully capture the biological complexity and functional diversity of the vaginal microbiome.

### 2.3. Host Factors Affecting the Vaginal Microbiome

The composition and stability of the VM are influenced by host factors such as age, hormonal fluctuations, behavior, and genetic predisposition [20,52,53,54]. Among these, age and hormonal status, particularly estrogen, play a central regulatory role. Estrogen promotes glycogen accumulation in vaginal epithelial cells, providing substrates for *Lactobacillus* fermentation and subsequent lactic acid production, thereby maintaining an acidic vaginal environment [55,56]. Prepubertal girls exhibit lower estrogen levels, with their VM primarily composed of *Staphylococcus epidermidis*, *Enterococci*, *E. coli* (aerobic microbe), *Peptococcus*, and *Peptostreptococcus* (anaerobic microbe) [54]. During the reproductive years, relatively high estrogen levels maintain a *Lactobacillus*-dominated microbiota with low diversity. However, with the decline of estrogen during menopause, *Lactobacillus* abundance decreases, microbial diversity increases, and the microbiota shifts toward dysbiosis [57,58]. Hormonal fluctuations during the menstrual cycle can also affect microbial dynamics. Menstrual blood raises vaginal pH, promoting anaerobic growth, and then restores *Lactobacillus* dominance after menstruation [59,60]. Pregnancy, characterized by sustained high levels of estrogen and progesterone, stabilizes the microbiota and promotes a protective, low-diversity profile [61,62,63].

Behavioral and lifestyle factors additionally influence microbial composition. Sexual activity, particularly with multiple partners, introduces external microbes and alters vaginal pH, potentially disrupting microbial balance [64,65,66]. Contraceptive methods exhibit varying effects: hormonal contraceptives often enhance *Lactobacillus* colonization, while the copper intrauterine device (copper IUD) correlates with increased microbial diversity [67,68,69]. Practices such as douching, the use of intravaginal antiseptics, and smoking are consistently linked to microbiota disruption and dysbiosis risk.

Ethnic and genetic differences are fundamental determinants of VM composition and stability. Population-based microbiome studies consistently demonstrate significant inter-ethnic variations. For example, approximately 80% to 90% of women of European and East Asian ancestry harbor *Lactobacillus*-dominated communities, with CST I (*L. crispatus*) specifically found in roughly 45% and 44% of these populations, respectively. In contrast, diverse, non-*Lactobacillus*-dominant communities (CST IV) are highly prevalent in women of African and Hispanic ancestry, observed in approximately 40% and 30% to 40% of these groups, compared to only 10% to 20% in White and Asian women [5,70].

While behavioral, socioeconomic, and environmental factors partially contribute to these disparities, accumulating evidence highlights the critical role of host genomic variation. Polymorphisms in genes encoding pattern recognition receptors (e.g., Toll-like receptors), pro-inflammatory cytokines (such as IL-1β and IL-6), and antimicrobial peptides have been associated with altered inflammatory responses to vaginal microorganisms and differential susceptibility to dysbiosis-related conditions, including bacterial vaginosis and vulvovaginal candidiasis [71]. Furthermore, genetic variants affecting mucin production, epithelial tight junction integrity, estrogen receptor signaling, and glycogen availability may further modulate microbial colonization efficiency, *Lactobacillus* adherence, and lactic acid production.

Understanding the VM is essential given its significant impact on female reproductive health. Despite advances in sequencing technologies and growing interest in microbiome diagnostics, challenges such as inter-individual variability and methodological inconsistencies persist. Variations in sampling, sequencing, and analysis limit comparability across studies, while the functional implications of specific microbial shifts are not fully understood. Deviations from *Lactobacillus* dominance do not necessarily mean that there is a health issue. These complexities highlight the importance of personalized and context-aware evaluations of vaginal microbiota in clinical research and practice. Such approaches are essential for improving our understanding of how these microbial communities influence health and disease.

## 3. Vaginal Microbiome Imbalance and Vaginitis

**Figure 2:**
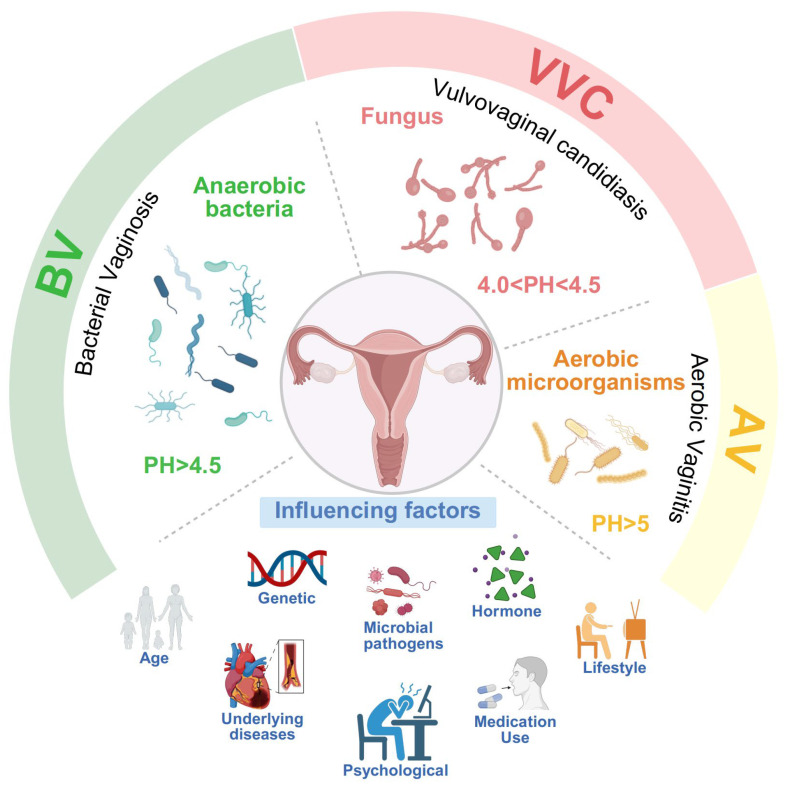
Microbial Characteristics and Influencing Factors in Infectious Vaginal Diseases. This figure illustrates the microbial pathogens and vaginal pH levels associated with different infectious vaginal diseases. It highlights the microbial features of BV, VVC and AV, while also emphasizing the various factors that influence vaginal health.

Vaginal dysbiosis promotes mucosal inflammation and barrier dysfunction, including epithelial remodeling and dysregulated immune responses [72,73]. These changes create a permissive niche for pathogen expansion and the development of vaginitis. Globally, over one billion women are affected by vaginal disorders, causing symptoms such as vaginal odor, irritation, burning sensations, pruritus, dysuria, and dyspareunia, which significantly burden reproductive health, psychological well-being, and social functioning [21,74]. Epidemiological studies indicate that among patients with vaginitis, BV accounts for approximately 40–50% of all cases, VVC for 20–25%, while AV is less common but is gaining increasing recognition and attention (Figure 2). Non-infectious forms of vaginitis, such as atrophic, irritative, and allergic vaginitis, comprise only 5–10% [75,76].

It is now widely accepted that BV, VVC, and other infectious vaginal diseases are not solely caused by isolated pathogens but rather result from broader disruptions of vaginal microecological structure and function. Thus, elucidating the shared and disease-specific mechanisms associated with VM dysbiosis is critical for advancing our understanding of disease pathogenesis and for developing more effective and targeted clinical interventions. The subsequent sections will review, by disease category, the microbiological characteristics and pathogenic mechanisms related to vaginal microbiota imbalance in BV, VVC and AV.

### 3.1. Bacterial Vaginosis (BV)

BV is the most prevalent reproductive tract disorder among women of reproductive age worldwide, affecting approximately 23–29% of women and imposing a substantial economic burden, with annual healthcare costs estimated at US$4.8 billion [34,77]. Marked disparities in BV prevalence have been reported across populations, with higher rates observed among Hispanic and Black women [78,79].

From a microbiome perspective, BV is characterized by a vaginal microbial community structure resembling CST IV. In women with BV, obligate anaerobes, such as *Prevotella* spp., *Atopobium vaginae*, *Sneathia* spp., and *Megasphaera* spp., along with facultative anaerobes like *Gardnerella* spp., are notably enriched, while the dominance of *Lactobacillus* is significantly reduced, resulting in a vaginal pH exceeding 4.5 [75,80,81]. Among these anaerobic bacteria, *G. vaginalis* is widely regarded as a key organism in BV pathogenesis, but it can also be detected in a subset of healthy women [82,83,84]. These findings indicate that BV arises not from colonization by a single pathogen but from a complex dysbiotic driven by strain-specific virulence, cooperative interactions within the microbial community, and altered host immunity.

BV is strongly linked to adverse reproductive outcomes, including preterm birth, infertility, pelvic inflammatory disease, and increased susceptibility to STIs [10,81,85,86,87,88,89]. Accumulating mechanistic evidence suggests that BV-associated anaerobic dysbiosis, combined with its metabolic byproducts, impairs endometrial function, disrupts embryo implantation, and increases the risk of pregnancy failure and miscarriage [90,91,92]. Concurrently, elevated vaginal pH and microbial imbalance have been shown to impair sperm motility and viability, thereby further compromising fertility [93,94].

Growing evidence indicates that bacterial biofilms play a critical role in the pathogenesis and persistence of BV. BV-associated microbial communities, particularly those dominated by *G. vaginalis*, form stable biofilms on the vaginal epithelium that enhance bacterial adhesion and resistance to host defenses and antimicrobial therapy [95,96]. Notably, similar biofilm structures have been detected in the fallopian tubes and endometrium, supporting the hypothesis that BV may facilitate ascending infection into the upper reproductive tract and contribute to adverse pregnancy outcomes [97]. Moreover, BV is associated with significantly elevated levels of pro-inflammatory cytokines in vaginal secretions, including interleukins and tumor necrosis factor-α (TNF-α) [97,98,99].

### 3.2. Vulvovaginal Candidiasis (VVC)

VVC is the second most common form of infectious vaginitis after BV, affecting approximately 75% of women at least once during their lifetime, with the highest incidence observed among women of reproductive age [41,100]. A total of 5–10% of women who experience an initial episode of VVC subsequently develop recurrent vulvovaginal candidiasis (RVVC), which is commonly defined as four or more episodes per year [29,101]. This recurrent disease significantly impairs quality of life, imposing long-term psychological and economic burdens [102,103,104]. The risk of VVC varies across populations and is influenced by multiple host and environmental factors, with higher prevalence reported among black women and in settings characterized by high estrogen exposure, frequent antibiotic use, and metabolic disorders such as diabetes [104,105].

Clinically, VVC is characterized by the excessive proliferation of *Candida* species, predominantly *C. albicans*, accompanied by a pronounced inflammatory response, such as vaginal pruritus, burning sensations, pain, erythema, edema, and abnormal vaginal discharge [100,106,107]. Based on clinical severity, causative species, and host status, VVC is classified into uncomplicated and complicated forms. Uncomplicated VVC typically affects immunocompetent women, is most often caused by *C. albicans*, and presents as mild to moderate disease with infrequent recurrence. In contrast, complicated VVC encompasses severe disease, infections caused by non-*albicans Candida* species, VVC occurring during pregnancy or in the context of underlying conditions such as poorly controlled diabetes or immunosuppression, as well as RVVC in otherwise immunocompetent women [108].

From a microbiological perspective, *C. albicans* is a commensal member of the normal vaginal microbiota and can asymptomatically colonize the vaginal cavity under homeostatic conditions. Among *Candida* isolates from women with VVC, approximately 80–92% are *C. albicans* [107]. The remaining cases are attributed to non-albicans *Candida* species, such as *C. glabrata*, *C. krusei*, *C. dubliniensis*, and *C. parapsilosis*, with *C. glabrata* being the most prevalent [107,109,110,111]. The pathogenicity of *C. albicans* is closely linked to its morphological plasticity, expression of virulence factors, and biofilm formation. The yeast-to-hypha transition of *C. albicans*, a key virulence determinant, requires specific innate immune signaling pathways [112]. A low vaginal pH inhibits this morphological shift, thereby constraining fungal pathogenicity [113]. Furthermore, *C. albicans* can promote fungal overgrowth and disrupt the vaginal epithelial barrier by forming biofilms and secreting virulence factors, such as secreted aspartyl proteases (SAPs) [104].

Most *Lactobacillus* species exert antifungal effects by maintaining an acidic environment, producing bioactive metabolites, competing for nutrients and adhesion sites, and modulating host immunity [73,114,115,116]. Yet women with VVC often show a vaginal microbiota composition similar to that of healthy individuals, with *Lactobacillus* species remaining predominant. This state is frequently characterized by an increased relative abundance of *L. iners*. These findings suggest that susceptibility to VVC may reflect functional impairment of *Lactobacillus*-mediated defense rather than a simple loss of *Lactobacillus* dominance [3].

Consistent with this concept, host immune responses play a central role in VVC pathogenesis. Women with symptomatic VVC display markedly increased vaginal neutrophil infiltration, which correlates positively with fungal burden yet fails to achieve effective fungal clearance [117]. This paradox supports the notion that VVC symptoms arise not from insufficient immune defense but from excessive or dysregulated innate immune activation. In contrast, non-*albicans Candida* species, such as *C. glabrata*, are frequently detected in both symptomatic and asymptomatic women and generally exhibit reduced virulence compared with *C. albicans* [107,118,119]. This observation underscores that VVC severity is not determined solely by fungal presence, but is critically shaped by the fungal species, host immune responses and the vaginal microenvironment.

Collectively, these insights indicate that VVC is a multifactorial disease driven by the complex interplay between fungal virulence, vaginal microecology, and host immune responses.

### 3.3. Aerobic Vaginitis (AV)

AV is an infectious vaginal disorder characterized by marked depletion of *Lactobacillus* species, the enrichment of aerobic or facultative anaerobic bacteria, and a pronounced inflammatory response with epithelial damage. Epidemiological studies indicate that the prevalence of AV in the general female population is approximately 7–12%, which is lower than that of BV [120]. However, AV occurs more frequently in pregnant, perimenopausal, and postmenopausal women, and is associated with adverse reproductive outcomes [48,121,122].

Compared with BV and VVC, AV is distinguished by robust innate immune activation, including extensive neutrophil infiltration, epithelial cell shedding, and elevated levels of pro-inflammatory cytokines, with the severity of inflammation correlating positively with mucosal damage [107]. These immune responses are closely linked to the observed alterations in microbial community structure.

Significantly elevated aerobic bacteria in patients with AV include *Enterococcus faecalis*, *E. coli*, GBS, and *Staphylococcus aureus* [123,124,125]. Among these, *E. faecalis* is the most frequently isolated species in AV, followed by *E. coli* and *S. agalactiae* [122,126]. These aerobic pathogens can disrupt the vaginal epithelial barrier and activate host immune responses through the production of toxins, proteases, and other pro-inflammatory factors. AV-associated *E. coli* strains are predominantly extraintestinal pathogenic variants, whose virulence traits are closely linked to adverse pregnancy outcomes [127,128,129]. In addition, *S. aureus*-derived Panton-Valentine leukocidin (PVL) can induce leukocyte necrosis and apoptosis, thereby amplifying local inflammatory responses [130].

Overall, the pathogenic mechanism of AV differs fundamentally from the anaerobe-dominated dysbiosis observed in BV and the fungal overgrowth characteristic of VVC, thereby further expanding the microecological spectrum of infectious vaginal diseases.

## 4. Vaginal Microbiota and Sexually Transmitted Infections (STIs)

In addition to common vaginitis, STIs remain a significant global public health burden that disproportionately impacts women of reproductive age. STIs are caused by a wide spectrum of pathogens, including bacteria (such as *Chlamydia trachomatis*, *Neisseria gonorrhoeae*, *Mycoplasma genitalium*, and *Treponema pallidum*), viruses (including HIV, HSV-1 and -2, HPV, Mpox, and hepatitis B and C viruses), and parasites (such as *Trichomonas vaginalis*, scabies, and lice) [131]. Increasing evidence indicates that susceptibility to STIs is not solely the result of pathogen exposure; they are also profoundly shaped by the ecological characteristics of the vaginal microenvironment and the composition of the vaginal microbiome [47,132,133]. This section summarizes current knowledge on the bidirectional interactions between the VM and major STIs, highlighting how microbial community structure influences infection acquisition, persistence, and disease outcomes.

### 4.1. Human Papillomavirus (HPV) Infection

Cervical cancer is the fourth most common cancer and can be caused by the most common STI among women worldwide. It accounts for over 600,000 new cases and more than 340,000 deaths annually, creating a disproportionate burden in low- and middle-income countries [134,135]. Persistent infection with high-risk HPV genotypes, particularly HPV-16 and HPV-18, is the principal driver of cervical carcinogenesis. While most HPV infections are transient and cleared spontaneously, a subset of women develop persistent infection that can progress to high-grade cervical lesions and malignancy. Increasing evidence suggests that the vaginal microbiome plays a crucial regulatory role in HPV infection persistence, host immune responses, and the progression of cervical cancer.

HPV infection and persistence are associated with distinct vaginal microbiota configurations rather than with uniform depletion of *Lactobacillus*. HPV-positive women frequently exhibit microbiota dominated by *L. iners* or characterized by increased microbial diversity with reduced representation of *L. crispatus* [136,137]. Longitudinal studies indicate that transitions from *L. crispatus* dominated communities toward *L. iners* dominant or non-*Lactobacillus* dominated states are associated with decreased rates of HPV clearance and increased risk of persistent infection [138]. These observations highlight that HPV-associated risk is linked to functional attributes of the vaginal microbiota rather than to microbial diversity alone.

Mechanistically, the vaginal microbiota influences HPV persistence by modulating local immune surveillance and epithelial signaling pathways. Research indicates that D-lactic acid, produced by *L. crispatus* and *L. jensenii*, inhibits YAP1 signaling and suppresses epithelial stem cell renewal, exerting protective anticancer effects. Conversely, the reduced production of D-lactic acid in *L. iners*-dominant communities has been associated with impaired antiviral immune responses and altered epithelial differentiation, potentially creating a permissive environment for viral maintenance [139]. *Streptococcus* can synthesize nucleic acids by utilizing environmental nucleotides. This process may deplete host nucleotide pools and inhibit anti-inflammatory metabolic pathways in *Lactobacillus*, such as adenosine synthesis [140]. Additionally, metabolites produced by non-*Lactobacillus* dominated communities through amino acid degradation can induce oxidative stress in cervical cells, accelerating the proliferation of abnormal cells.

Importantly, the relationship between HPV and the vaginal microbiome is bidirectional. While vaginal microbiota instability promotes HPV acquisition and persistence, HPV infection itself actively disrupts microbial homeostasis. Mechanistically, the HPV E7 oncoprotein interferes with host NF-κB and Wnt/β-catenin signaling pathways, resulting in downregulation of host defense peptides (such as SLPI and HβD1) essential for *Lactobacillus* colonization and survival. This selective depletion of *Lactobacillus* further exacerbates microbial imbalance and reinforces a dysbiotic microenvironment [141]. Together, these HPV-induced microbial alterations create a permissive niche that supports viral persistence, chronic inflammation, and progressive epithelial dysregulation, accelerating the transition from precancerous lesions to invasive cervical cancer.

### 4.2. Human Immunodeficiency Virus (HIV) Infection

HIV infection remains a leading cause of death among women of reproductive age, with a significant proportion (26%) occurring among young women in sub-Saharan Africa. Vaginal mucosal transmission represents the primary route of HIV acquisition in women [142,143]. To establish infection, HIV must penetrate the vaginal mucosal barrier to directly infect epithelial CD4+ T cells or be internalized by antigen-presenting cells within the epithelium before being presented to HIV-susceptible cells [144].

*Lactobacillus*-dominant communities and their metabolites can limit mucosal inflammation, reduce the recruitment and activation of HIV target cells, and collectively lower the risk of infection. Moreover, microbiome-derived metabolites also impact mucosal barrier integrity. For instance, metabolites derived from a *Lactobacillus*-dominant vaginal microbiome help preserve epithelial barrier integrity against HIV. In contrast, metabolites produced by dysbiotic-associated bacteria such as *Prevotella* and *Gardnerella*—particularly short-chain fatty acids including acetic acid, succinic acid and butyric acid—have been shown to disrupt epithelial tight junctions and increase HIV translocation across the mucosa [145]. Additional studies indicate that suboptimal vaginal microbiota may impair epithelial immune cell function, thereby promoting epithelial damage and facilitating HIV presentation to T cells [146]. Notably, research by Teijlingen et al. demonstrates that *Prevotella timonensis* enhances HIV-1 uptake by vaginal Langerhans cells and confers resistance to HIV-1 prophylactic interventions, thereby increasing susceptibility to HIV-1 infection [147].

In summary, these findings emphasize the pivotal role of the vaginal microbiome in shaping mucosal barrier integrity, immune activation, and cellular susceptibility, underscoring vaginal dysbiosis as a critical biological cofactor in HIV transmission.

### 4.3. Herpes Simplex Virus (HSV) Infection

HSV infections are widespread globally, with HSV-2 transmitted almost exclusively through sexual contact, causing genital herpes. In 2016, approximately 491.5 million (13%) individuals aged 15 to 49 worldwide were infected with HSV-2, with adolescent females accounting for the highest number of new infections [148]. Epidemiological studies have identified several behavioral risk factors for HSV infection, including the use of progestin-only contraceptives, multiple sexual partners, and vaginal douching [149].

From a microecological perspective, HSV-2 infection is frequently associated with vaginal microbial communities characterized by reduced protective capacity and increased epithelial vulnerability. Women who are HSV-2-positive more frequently exhibit vaginal microbiota enriched with *G. vaginalis*. In addition, higher Nugent scores—a Gram stain-based diagnostic scoring system for BV—have been associated with an increased risk of HSV acquisition [47,150]. Mechanistic studies suggest that *G. vaginalis* can facilitate HSV infection by compromising epithelial barrier integrity and attenuating mucosal immune defenses, thereby creating a permissive environment for viral entry and replication [151].

Conversely, experimental evidence indicates that *Lactobacillus* species can exert antiviral effects through immune-modulatory mechanisms rather than direct microbial competition. Cell surface components of *Lactobacillus brevis* have been shown to inhibit HSV-2 proliferation [152], while extracellular vesicles derived from *Lactobacillus rhamnosus* activate the NOD2–type I interferon signaling pathway and induce interferon-stimulated gene expression, thereby enhancing antiviral host responses [153]. Together, these findings suggest that the vaginal microbiota influences HSV susceptibility primarily by shaping local immune tone and epithelial resilience, highlighting HSV infection as a microecologically sensitive, immune-modulated viral disease.

### 4.4. Chlamydia trachomatis (CT) Infection

CT is the most common bacterial STI pathogen globally, accounting for over 130 million cases worldwide in 2019, and has continued to rise in incidence over the past 40 years; vagina and cervix are the most common sites of infection in women [154]. While female urogenital CT infections are typically asymptomatic, untreated cases may lead to various sequelae and complications [155].

The vaginal environment of CT-positive women typically exhibits a CST IV-A patter [154,156]. Studies indicate that BV patients face a 62% increased risk of developing CT infections [154]. The BV-associated microbiota creates a microenvironment characterized by low acidity and low oxygen, thereby diminishing the inhibitory effect of Interferon-γ (IFN-γ) on *Chlamydia* growth [157,158]. Additionally, CT is a tryptophan nutrient-deficient organism. Certain members of the BV microbiome can assist *Chlamydia* in synthesizing tryptophan by producing indole, thereby promoting its survival and growth [159]. In contrast, *Lactobacillus*-dominated microbiomes inhibit CT infection by eliminating tryptophan. [160,161]. Further mechanistic studies revealed that *L. crispatus* interacts with cervical epithelial cells and alters membrane lipid composition [116]. It also reduces the surface expression of α5β1 integrin. As a result, α5β1-mediated adhesion and invasion of CT are inhibited, leading to reduced infectivity. *Candidatus* Lachnocurva vaginae possesses a D-lactate dehydrogenase gene. By metabolizing D-lactate, it may diminish the protective effect of D-lactate against CT infection, thereby increasing host susceptibility to CT [162]. These findings offer new perspectives for controlling chlamydial infections and may contribute to the development of novel diagnostic or prognostic tools.

### 4.5. Trichomonas vaginalis Infection

Trichomoniasis, caused by the flagellated protozoan *Trichomonas vaginalis*, is the most prevalent non-viral STI worldwide, with an estimated 156 million new cases annually [163]. Increasing evidence indicates that *T. vaginalis* does not act alone during infection but instead engages in complex and dynamic interactions with the vaginal microbiome.

Epidemiological and sequencing-based studies consistently demonstrate a high degree of overlap between *T. vaginalis* and BV-like microbiota configurations, with frequent co-occurrence of *Gardnerella*, *Atopobium*, *Prevotella*, and other BV-associated anaerobes. Hinderfeld et al. demonstrated that these anaerobic bacteria cooperate with *T. vaginalis* to disrupt tight junction complexes in the cervicovaginal epithelium, leading to increased paracellular permeability and enhanced protozoan adhesion to epithelial cells [164]. Moreover, the biofilm they form further facilitate *T. vaginalis* attachment to host cells, thereby amplifying its pathogenic effects [165].

Beyond physical barrier disruption, dysbiosis also amplifies the inflammatory cascade associated with *T. vaginalis*. For example, *Prevotella bivia* has been shown to enhance expression of the *T. vaginalis* adhesion gene *ap65*, increase protozoan cytotoxicity, and promote the secretion of pro-inflammatory mediators, including IL-6, IL-8, CXCL1, and IP-10, in cervical epithelial cells [166]. Together, these findings highlight a synergistic relationship between *T. vaginalis* and the dysbiotic vaginal microbiota, in which microbial community imbalance facilitates protozoan adhesion, epithelial barrier disruption, and inflammatory amplification, thereby promoting disease severity and persistence.

Overall, interactions between the VM and STIs are highly pathogen-specific rather than driven by generalized dysbiosis alone. Different STI pathogens exploit distinct microbiota-associated host pathways to establish persistence. At the same time, VM dysbiosis increases susceptibility to STI. Once infection occurs, it can further destabilize vaginal microbial communities, reinforcing microecological imbalance.

## 5. Vaginal Microbiota and Non-Infectious Gynecological Conditions

Menopause represents a major physiological transition in a woman’s life, characterized by declining estrogen levels and elevated gonadotropins such as follicle-stimulating hormone (FSH) and luteinizing hormone (LH) [167]. These hormonal alterations significantly affect the anatomy and function of urogenital tissues and may lead to vulvovaginal atrophy (VVA). In 2014, the North American Menopause Society and the International Society for the Study of Women’s Sexual Health introduced the term genitourinary syndrome of menopause (GSM) to more comprehensively describe VVA and its associated genital, sexual, and urinary manifestations [168,169]. The symptoms of GSM include genital discomfort (dryness, burning, and irritation), sexual dysfunction (dyspareunia and postcoital bleeding), urinary discomfort, urgency, nocturia, incontinence, and recurrent urinary tract infections [170].

Unlike infectious vaginitis, GSM is not caused by pathogenic invasion but reflects sustained alterations in vaginal tissue biology and microecological function driven by hormonal deprivation. While estrogen levels decline universally after menopause, only a subset of women develop GSM, indicating that additional biological factors contribute to disease susceptibility and symptom heterogeneity. Estrogen deprivation disrupts epithelial and immune homeostasis, leading to functional dysbiosis [171]. As a consequence, the postmenopausal vaginal microbiota frequently exhibits reduced *Lactobacillus* abundance, increased microbial diversity, and community structures resembling CST IV-C, although without the overt pathogen overgrowth characteristic of infectious vaginitis [6]. Estrogen deprivation also leads to epithelial thinning, reduced mucus production, and impaired epithelial turnover, collectively weakening barrier integrity and increasing tissue sensitivity to mechanical and chemical stimuli [172]. In parallel, changes in mucosal immune regulation and low-grade inflammatory signaling further disrupt host–microbiota crosstalk and hinder re-establishment of a stable, protective microbial community. Compared to traditional antimicrobial therapies, interventions targeting upstream host factors—such as local estrogen replacement and strategies to restore epithelial integrity—are more effective in alleviating symptoms and promoting both microecological and microbiome recovery [173]. Persistent GSM-associated microecological instability may also increase susceptibility to secondary infections or impair microbiota restoration following antimicrobial exposure, underscoring the broader implications of GSM for vaginal health across the lifespan [13].

Beyond hormone deficiency-associated conditions such as GSM, emerging evidence suggests that vaginal microbiome dysbiosis may also be implicated in endocrine-related disorders, including polycystic ovary syndrome (PCOS) [14]. Reduced *Lactobacillus* dominance and increased microbial diversity have been observed in women with PCOS compared with healthy controls [174]. Menstrual irregularity and abnormal hormonal profiles—particularly hyperandrogenism and altered estrogen levels—are considered major contributors to these microbial alterations [175]. Although causal relationships remain to be established, these findings suggest that endocrine–metabolic disturbances may interact bidirectionally with vaginal microbial ecology.

Collectively, conditions such as GSM and PCOS exemplify host-driven forms of vaginal dysbiosis, in which upstream hormonal and epithelial alterations, rather than primary microbial pathogenicity, initiate functional perturbations of the vaginal ecosystem. This host-centered framework provides a conceptual contrast to pathogen-driven dysbiosis observed in infectious vaginitis and STIs.

## 6. Vaginal Microbiota in Clinical Practice: Diagnostic Techniques and Therapeutic Strategies

Accumulating evidence demonstrates that the VM plays a central role in maintaining vaginal health and shaping susceptibility to a wide spectrum of gynecological conditions. Alterations in microbial community structure, metabolic activity, and host–microbe interactions underlie both infectious and non-infectious vaginal disorders, highlighting the clinical importance of accurately characterizing the VM and translating microbiome insights into effective therapeutic strategies. In recent decades, advances in diagnostic technologies and microbiome-based interventions have substantially reshaped current concepts of vaginal disease management, shifting the paradigm from pathogen-centered treatment toward microecology-informed and personalized care.

### 6.1. Diagnostic Techniques for Vaginal Microbiota Characterization

Traditional diagnostic approaches for diagnosing vaginal diseases—including vaginal smear microscopy, microbial culture, vaginal pH measurement, and the Nugent scoring system—remain widely used in routine clinical practice due to their simplicity, low cost, and rapid turnaround [81,176]. Wet mount microscopy and Gram staining allow for the evaluation of microbial morphology, leukocyte infiltration, and epithelial integrity, and are commonly applied in the diagnosis of BV, AV, and trichomoniasis [177,178,179]. However, these methods are limited by operator dependency, reduced sensitivity for fastidious or uncultivable microorganisms, and an inability to capture the complexity of microbial community structure and function [9,180,181,182].

The limitations of conventional methods have driven the adoption of molecular techniques, marking a paradigm shift from phenotypic observation to genotypic identification. Targeted approaches such as polymerase chain reaction (PCR) enable sensitive and reproducible detection of specific pathogens or key bacterial taxa, thereby improving diagnostic accuracy. High-throughput sequencing of the 16S rRNA gene and internal transcribed spacer (ITS) regions has further enabled comprehensive profiling of vaginal microbial communities, facilitating the identification of CSTs and revealing that many vaginal disorders arise from community-level dysbiosis rather than single-pathogen infection [84].

To achieve higher taxonomic resolution and functional insight, shotgun metagenomic sequencing has emerged as a powerful tool for vaginal microbiome research. This approach enables species- and strain-level identification while simultaneously inferring functional potential, thereby providing deeper insights into disease-associated microbial shifts and host–microbe interactions [24,183,184]. However, metagenomic sequencing may be limited by insufficient sequencing depth for low-abundance taxa and, when used alone, cannot fully capture microbial activity or host–microbe functional interactions. However, metagenomic sequencing remains constrained by limited sensitivity for low-abundance taxa and, when used alone, cannot fully capture real-time microbial activity or host–microbe functional dynamics. Importantly, taxonomic composition does not necessarily reflect metabolic output, virulence expression, or ecological interactions within the vaginal ecosystem.

To overcome these limitations, multi-omics integration has emerged as a promising frontier in vaginal microbiome research [19,185]. Metatranscriptomics enables functional-level analysis by profiling actively expressed microbial genes, thereby revealing functional differences between healthy and diseased states [186]. Metabolomics serves as a critical bridge between microbial community structure and host immune responses by systematically characterizing small-molecule metabolites that mediate functional host–microbe interactions [187]. Proteomics further complements these approaches by identifying microbial enzymes, virulence factors, and host immune-related proteins involved in disease development and progression [19]. Nevertheless, sequencing- and omics-based methodologies remain subject to technical variability. Differences in sampling site selection, menstrual cycle timing, specimen handling, nucleic acid extraction efficiency, sequencing depth, and bioinformatic pipelines can introduce systematic bias, contributing to inconsistencies in CST classification and microbial abundance estimates across studies.

**Figure 3:**
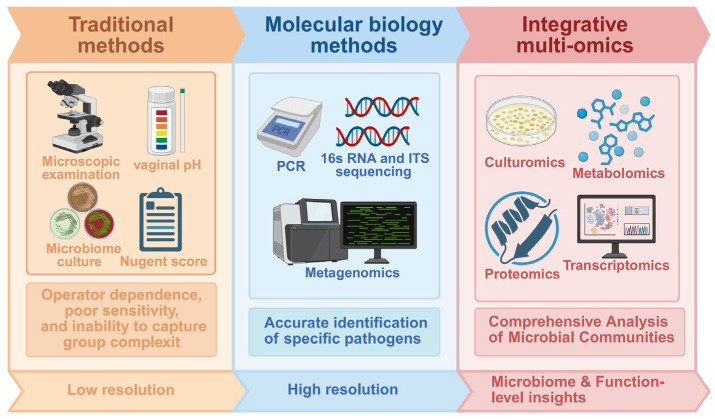
Diagnostic Techniques for Vaginal Diseases. This figure illustrates the various diagnostic techniques used to assess vaginal diseases, ranging from traditional methods with low resolution, such as microscopic examination, microbiome culture, and vaginal pH measurement, to molecular biology techniques like PCR, 16S rRNA, and ITS sequencing, which enable accurate identification of specific pathogens. It also integrates omics approaches, including metagenomics, metatranscriptomics, metabolomics, and the emerging field of culturomics, providing comprehensive insights into microbial community structure and function. These advanced methods allow for a more detailed and personalized understanding of vaginal health and disease.

In this context, culturomics provides a complementary and indispensable strategy by enabling the systematic isolation of viable microbial strains. Through the application of diverse and optimized culture conditions, followed by identification using matrix-assisted laser desorption/ionization–time-of-flight mass spectrometry (MALDI-TOF MS) or full-length 16S rRNA and ITS gene sequencing, culturomics facilitates the discovery and characterization of previously unrecognized or low-abundance microorganisms [188]. These organisms are often referred to as the “dark matter” of the microbiome. Importantly, culturomics not only expands microbial reference genome databases but also supplies indispensable live strain resources for mechanistic studies, including host–microbe interaction modeling, in vitro and in vivo experimentation, and the development of next-generation probiotic therapeutics. Figure 3 illustrates the different diagnostic techniques, from traditional methods to advanced molecular and multi-omics approaches.

### 6.2. Microbiome-Dependent Therapeutic Strategies for Vaginal Diseases

Currently antimicrobial agents remain the cornerstone of vaginitis treatment. For BV, the standard treatment regimen includes oral or vaginal administration of metronidazole or clindamycin [189]. AV management requires individualized approaches guided by antibiotic susceptibility profiles [190]. Trichomoniasis treatment primarily relies on a short-course of oral metronidazole, with tinidazole as an alternative [191,192]. For uncomplicated VVC, current treatment guidelines recommend oral fluconazole, while complicated cases typically require prolonged topical azole therapy or multiple-dose oral fluconazole regimens [193,194,195].

While these agents effectively target pathogens and alleviate symptoms, their non-selective antimicrobial activity disrupts both harmful and beneficial microbiota, compromising vaginal microbial homeostasis [15,196]. This dysbiosis often results in persistent imbalances and high recurrence rates [16]. Therefore, there is growing consensus that effective management of vaginal infections requires a shift from broad-spectrum antimicrobial treatments to more precise, microbiome-based interventions [197].

In this context, probiotic lactobacilli, owing to their safety profile, are increasingly explored as alternatives or adjuncts to antibiotics to mitigate dysbiosis and antimicrobial resistance associated with excessive antibiotic use, while promoting pathogen control [198].

Currently, probiotics have emerged as a promising adjuvant or alternative to traditional antibiotic treatment [17]. For example, Recine et al. reported that patients who received vaginal tablets containing probiotics after metronidazole treatment exhibited reduced recurrence rates of BV and improved vaginal pH levels [199]. Mändar et al. demonstrated that both oral and vaginal administration of *L. crispatus* capsules over three months significantly improved the abundance of vaginal *Lactobacillus* and alleviated clinical symptoms in patients with BV and VVC [200]. Similarly, Heczko et al. evaluated an oral probiotic preparation combined with standard metronidazole therapy and found a significant reduction in recurrence rates, alongside sustained low vaginal pH and Nugent scores [201].

Importantly, several ongoing and recently completed randomized controlled trials (RCTs) are evaluating *Lactobacillus*-based live biotherapeutic products (LBPs). For instance, recent phase IIb trials investigating specific *L. crispatus* strains (such as LACTIN-V) have demonstrated significant reductions in BV recurrence compared to placebo, highlighting their clinical potential [202].

Despite promising results, probiotic supplementation via oral or vaginal routes has yielded inconsistent clinical outcomes [203]. This may be due to heterogeneity in probiotic strains, dosages, treatment regimens, and inter-individual differences in colonization stability [204,205]. Furthermore, translating these probiotics into approved routine therapies faces significant regulatory challenges. As live biotherapeutic products, they must meet stringent regulatory requirements regarding strain characterization, manufacturing consistency, and viability over time. The absence of harmonized regulatory frameworks highlights the need for rigorously designed Phase III trials to firmly establish their long-term efficacy and safety. Novel interventions for the VM are shown in Table 2.

To overcome the colonization limitations and limited ecological impact of single-strain probiotics, Vaginal Microbiota Transplantation (VMT) has emerged as a more comprehensive restoration strategy. VMT involves the transfer of entire vaginal microbial communities from healthy donors to affected recipients, thereby increasing the abundance of beneficial bacteria and enhancing microbial competition against pathogenic species [217]. Donor-derived *Lactobacillus* strains may outcompete pathogenic microorganisms for nutrients and ecological niches, reducing pathogen abundance below disease-triggering thresholds and promoting long-term microbial stability.

In a noteworthy study by Lev-Sagie et al. [18], vaginal secretions from three healthy donors were introduced into five patients with refractory BV. Most recipients exhibited no signs of recurrence for up to 21 months following the last transplant, highlighting the potential of VMT in managing persistent cases. Additionally, Li et al. assessed VMT treatment in *G. vaginalis*-induced BV mouse models, finding that VMT reduced bacterial loads and pro-inflammatory cytokine secretion, effectively suppressing NF-κB activation [218].

Despite its therapeutic potential, VMT is not without risks. A major concern is the potential transmission of pathogenic or opportunistic microorganisms from donor material. At present, standardized protocols for donor screening, transplantation procedures, and long-term post-transplantation monitoring are lacking. Consequently, clinical applications of VMT have largely been limited to refractory cases of BV and may require repeated administrations to achieve sustained efficacy. Under these circumstances, comprehensive risk assessment and informed consent should be regarded as essential prerequisites prior to clinical implementation.

Increasing evidence suggests that a “one-size-fits-all” approach to the treatment of vaginitis is insufficient, given the substantial inter-individual variability in vaginal microbiota composition, host immune responses, and treatment outcomes [219]. Personalized and precision microbiota-based therapies therefore represent a promising future direction in the management of vaginal diseases.

Beyond taxonomic composition, the functional characteristics of the vaginal microbiome are increasingly recognized as critical determinants of disease susceptibility and therapeutic response. Multi-omics approaches enable comprehensive characterization of microbial metabolic activity, virulence potential, and host–microbe interactions [140,186,220]. Integration of these datasets may facilitate the identification of functional biomarkers, such as lactic acid production, biofilm-associated pathways, or inflammatory mediators, which could be used to accurately predict treatment efficacy and recurrence risk [186,221,222].

Advances in vaginal microbiome profiling have enabled more precise definition of microecological states while exposing the limitations of pathogen-centered therapies. Together, these insights support a shift toward microbiome-dependent, precision-oriented interventions that prioritize restoration of microbial function and host–microbiota homeostasis to achieve durable vaginal health.

## 7. Future Perspectives

Despite rapid advances in vaginal microbiota research, most current knowledge remains largely descriptive, with associations between microbial community states and gynecological diseases often inferred from cross-sectional studies. The field must now transition from correlative frameworks to establishing causal relationships that explain how specific microbial functions, host pathways, and environmental factors jointly drive disease. Addressing this gap will require carefully designed longitudinal cohorts, intervention-based studies, and mechanistic validation in experimental models that reflect the complexity of the vaginal ecosystem.

A critical priority for future research is the functional characterization of vaginal microbiota beyond taxonomic composition. While community state types have provided a useful descriptive framework, they do not fully capture microbial metabolic activity, virulence potential, or host–microbe interactions. Integrating multi-omics approaches with culture-based methods will be essential for identifying functionally relevant microbial traits, such as biofilm formation, metabolite production, and immune-modulatory capacity, and for determining how these features influence host physiology and disease susceptibility.

Furthermore, distinguishing between pathogen-driven and host-driven dysbiosis remains a critical challenge. The evidence reviewed in this article highlights that infectious vaginitis, sexually transmitted infections, and non-infectious gynecological conditions arise through distinct but overlapping ecological mechanisms. Future studies should aim to define disease-specific microecological signatures that incorporate host factors, including hormonal status, epithelial integrity, and immune responsiveness, rather than relying solely on microbial abundance profiles. Such an approach will be critical for improving disease stratification and for avoiding oversimplified interpretations of dysbiosis.

From a translational perspective, advancing vaginal microbiome research will require the development of predictive biomarkers that can guide personalized intervention strategies. Biomarkers derived from microbial function, host response, or their interaction may enable risk prediction, treatment selection, and monitoring of therapeutic efficacy. However, robust clinical validation and standardization will be necessary before such tools can be incorporated into routine practice.

Finally, realizing the full clinical potential of vaginal microbiota science will depend on interdisciplinary collaboration across microbiology, immunology, reproductive endocrinology, gynecology, and computational biology. By integrating ecological theory with mechanistic and clinical research, the field is shifting from reactive, pathogen-centered management toward proactive, precision-based modulation of the vaginal ecosystem. Such a shift represents not merely a technological advancement, but a conceptual redefinition of how vaginal health and disease are understood and managed.

## 8. Conclusions

The vaginal microbiome is increasingly recognized as an integral component of female reproductive and gynecological health. Vaginal health is determined not only by microbial composition but also by dynamic interactions among microbial function, epithelial integrity, hormonal regulation, and host immune responses. Disruption of this balanced microecological system contributes to a wide range of gynecological conditions, including infectious vaginitis, sexually transmitted infections, and non-infectious disorders such as GSM and PCOS. Importantly, vaginal dysbiosis represents a heterogeneous and context-dependent state, with pathogen-driven mechanisms predominating in some diseases and host-driven alterations underlying others. Recent progress in microbiome profiling, multi-omics integration, and functional validation has substantially improved the resolution at which vaginal microecology can be characterized, while also revealing the limitations of conventional pathogen-targeted therapies. Looking forward, the integration of mechanistic insights with refined diagnostic tools and microbiome-dependent, stratified intervention strategies is expected to advance personalized approaches for the prevention and management of vaginal disorders and to promote durable improvements in women’s health.

## Figures and Tables

**Table 1:** Relationship between Vaginal Microbiome and Various Vaginal Diseases.

Vaginal Microbiome	BV	VVC	AV	TV	CT	HPV	HIV	HSV	References
*Aerococcus*	↑	↑	-	-	↑	↓	-	-	[21]
*Atopobium*	↑	↑	-	-	↑	-	↑	-	[21,34,35,36,37]
*Bacteroides*	↑	↑	-	-	↑	-	-	-	[21]
*Bifidobacterium*	↑	↑	-	-	↑	↓	-	-	[10]
*Candida*	-	↑	-	-	-	-	-	-	[38,39]
*Clostridium*	↑	↑	-	-	↑	-	-	-	[21,40]
*Dialister*	↑	↑	-	-	↑	-	-	-	[21,40,41,42]
*Escherichia*	↑	↑	↑	-	↑	-	-	-	[21]
*Gardnerella*	↑	↑	-	↓	↑	↑	↑	↑	[3,10,12,36,37,39,41,43,44,45,46,47]
*Klebsiella pneumoniae*	-	-	↑	-	-	-	-	-	[48]
*Lactobacillus*	↓	↓	-	↓	↓	↓	↓	↓	[3,21,39,45,46,47]
*L*. *crispatus*	↓	↓	↓	-	↓	-	↓	-	[3,38,39,46,49,50]
*L*. *gasseri*	↑	↑	-	-	-	-	-	-	[3,38]
*L*. *iners*	↓	↑	-	↓	↑	-	-	↑	[3,38,47,49,50]
*L. johnsonii*	↑	-	↓	-	-	-	-	-	[3]
*Megasphaera*	↑	↑	-	-	↑	-	-	-	[3,50]
*Mobiluncus*	↑	-	-	-	-	-	-	-	[10,40,41]
*Mycoplasma*	-	-	-	↑	-	-	-	-	[12,42]
*Peptoniphilus*	↑	-	-	↑	-	-	-	-	[3,42]
*Peptostreptococcus*	↑	↑	-	-	-	-	-	-	[21]
*Prevotella*	↑	↑	-	↑	↑	-	-	-	[12,21,36,37,39,41,42,50]
*Pseudomonas putida*	-	-	↑	-	-	-	-	-	[48]
*Roseburia*	↑	↑	-	-	↑	-	-	-	[12,21]
*Sneathia*	↑	↑	-	↑	↑	↑	-	-	[10,40,41,51]
*Staphylococcus aureus*	-	-	↑	-	-	-	-	-	[48]
*Streptococcus*	↑	↑	↑	↑	-	↑	-	-	[3,39,42,51]

For each disease listed, microbial abundance trends are based on pooled data from multiple studies. Abbreviations: BV, bacterial vaginosis; VVC, vulvovaginal candidiasis; AV, aerobic vaginitis; TV, *Trichomonas vaginalis* infection; CT, *Chlamydia trachomatis* infection; HPV, human papillomavirus; HIV, human immunodeficiency virus; HSV, herpes simplex virus. **↑**: Indicates an increase in abundance. **↓**: Indicates a decrease in abundance. -: Indicates no significant change in abundance.

**Table 2:** Summary Table of Probiotic Therapy Outcomes in Human Studies.

Probiotics	Disease	Mechanism of Action	Treatment Method	Treatment Outcomes	References
*L. crispatus*	BV	Reduce IL-1α and soluble E-cadherin (a biomarker of epithelial barrier disruption) concentrations.	Oral probiotic gel after treatment with vaginal metronidazole.	Significantly lower incidence of recurrence of bacterial vaginosis.	[200,206]
	VVC	*L. crispatus* treatment could modulate the vaginal microbiome.	Oral or vaginal probiotic capsules.	Significantly increased the lactobacilli counts in their vagina, lowered the combined score of amount of discharge and itching/irritation.	[200]
	HPV	*L. crispatus* treatment could modulate vaginal and gut microbiota.	Oral administration for 12 months.	A higher percentage of clearance of PAP smear abnormalities in patients who took long-term oral *L. crispatus* M247.	[207]
*L. gasseri* DSM 14869	BV	Producing a thick (40 nm) EPS layer and hydrogen peroxide.	Daily vaginal administration of capsules following clindamycin therapy.	Eliminating the symptoms and improving the antibiotic treatment of BV.	[208,209]
*L. plantarum* P17630	VVC	Adhering to human vaginal cells thereby interfering with adherence of *C*. *albicans.*	Following conventional treatment with clotrimazole, intravaginal administration of *L. plantarum* P17630.	The number of *Lactobacillus* vaginalis significantly increased, physiological pH levels stabilized more effectively, and symptoms such as burning or itching showed marked improvement.	[210]
*L. rhamnosus* DSM 14870	BV	Producing SpaCBA pili and a 20 nm EPS layer, and inhibiting the growth of *G*. *vaginalis.*	Daily vaginal administration of capsules following clindamycin therapy.	Aggressive treatment of the patient with antibiotics combined with *Lactobacillus* administration can provide a long-lasting cure.	[208,209]
*L. rhamnosus* Fiti	HIV	Delay the decline of CD4 lymphocytes.	Consume yogurt supplemented with *L. rhamnosus* Fiti.	Delay the decline in CD4 lymphocytes, reduced inflammation and infection.	[211]
*L. rhamnosus* BMX 54	HPV + BV	Restoring a stable microbiota to eubiosis to curb viral infections.	Receiving standard BV treatment plus vaginal administration of *L. rhamnosus* BMX 54.	Compared with the short-term treatment group (3 months), the long-term treatment group (6 months) demonstrated a significantly higher HPV clearance rate.	[212]
*L. fermentum* 57A, *L. gasseri* 57C and *L. plantarum* 57B	AV + BV	Adhesion to human Caco-2 intestinal cells and A431 vaginal cell lines, reducing pathogen adhesion.	Oral co-administration of multispecies-lactobacilli with metronidazole.	Lengthening the relapse significantly and maintaining the acidity of vaginal pH.	[201]
*L. acidophilus* GLA-14, *L. rhamnosus* HN001	VVC	Producing antimicrobial substances like lactic acid, hydrogen peroxide and bacteriocin.	Oral probiotic capsule daily.	Itching and discharge showed significant improvement, with a marked reduction in recurrence rates.	[213]
*L. rhamnosus* GG, *L. pentosus* KCA1 and *L. plantarum* WCFS1	VVC	Inhibits the growth of *Candida* and its adhesion to epithelial cells.	Using a vaginal gel containing lactobacilli once daily before bedtime over the course of 10 days.	45% of women did not require rescue medication (3 × 200 mg fluconazole).	[214]
*L. acidophilus* LA-5 and *Bifidobacterium animalis* subsp. *lactis* BB-12	HIV	Correct dysbiosis and downregulate inflammation severity.	Take one capsule orally three times daily starting from the first day of radiation therapy.	Reducing the incidence and severity of radiation-induced diarrhea in cervical cancer patients	[215]
*L. crispatus* LBV88, *L. rhamnosus* LBV96, *L. gasseri* LBV150N and *L. jensenii* LBV116	*Ureaplasma parvum*	Secretes lactic acid and bacteriocins, creating an acidic vaginal environment.	Participants took one sachet a day of a probiotic supplement for a period of four weeks.	The relative abundance of *U. parvum* was significantly reduced in the intervention group.	[216]

## Data Availability

Not applicable.
